# Supplementary material for: Ethylene promotes SMAX1 accumulation to inhibit arbuscular mycorrhiza symbiosis
Source: Nat Commun. 2025 Feb 27;16:2025. doi: 10.1038/s41467-025-57222-w (PMC11868565; doi:10.1038/s41467-025-57222-w)
Supplement: Supplementary file 1 — Supplementary Information [file 41467_2025_57222_MOESM1_ESM.pdf]

Ethylene promotes SMAX1 accumulation to inhibit arbuscular mycorrhiza symbiosis

Das, Varshney *et al.*

This PDF file includes:
Supplementary Figures S1 to S13
Supplementary Tables S1 to S5
Supplementary References

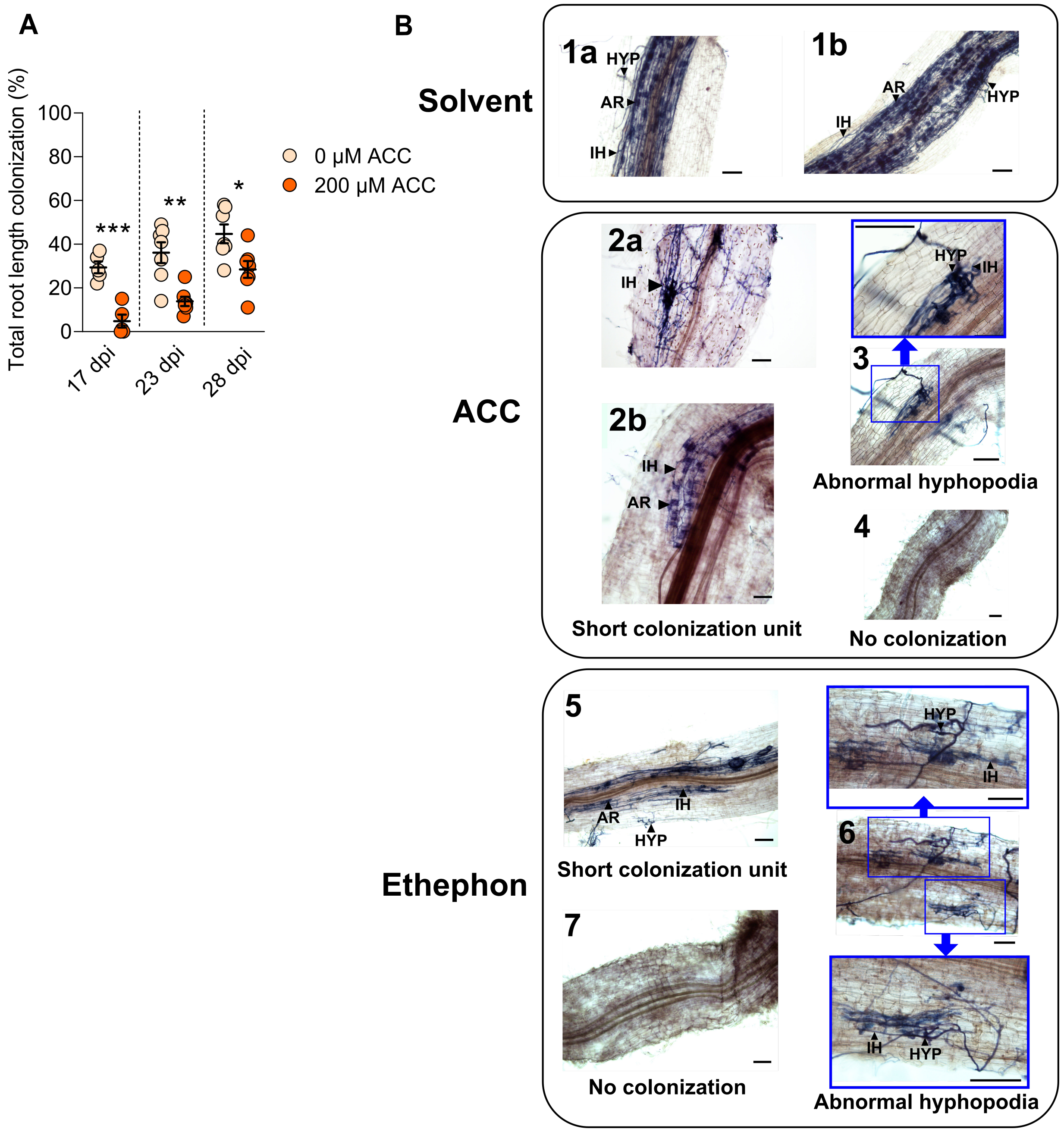

Supplementary Figure S1. Time course of ethylene-mediated suppression of AM development and morphological features of *Rhizophagus irregularis* colonization units upon ethylene treatment. A Percent total root length colonization of *L. japonicus* wild type co-cultured with *R. irregularis* for indicated time periods and treated with 200 µM ethylene, a precursor of ACC. Individual data-points and mean ± SE are shown. N=7; Mixed-effects model (REML) test with Sidak's multiple comparisons test [For time, F (1.971, 19.71) = 13.97; for treatment, F (1, 12) = 37.63 and for interaction, F (2, 20) = 0.7140]. Asterisks denote significance: * p ≤ 0.05; **p ≤ 0.01; *** p ≤ 0.001. **B** Bright field microscopy images of roots stained with acid-ink to visualize colonization of *L. japonicus* wild type colonization by *R. irregularis* at 4 wpi after treatment with solvent (water), 200 µM ACC or 100 µM ethephon. Scale bars, 100 µm. Abbreviations: HYP: hyphopodium; IH, intraradical hypha; AR, arbuscule.

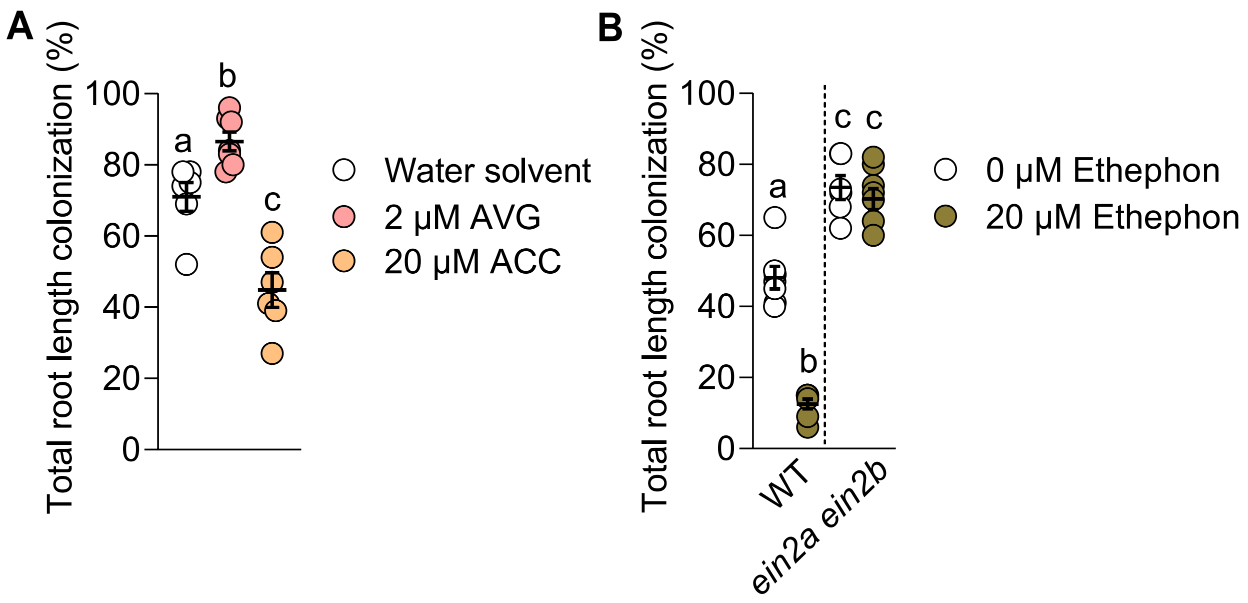

Supplementary Figure S2. Effect of ethylene signaling on AM development in *Lotus japonicus* roots in hydroponics. A Percent root length colonization of *L. japonicus* wild type co-cultured with *R. irregularis* for 4 weeks and treated with the ethylene biosynthesis inhibitor, AVG or the ethylene precursor, ACC at indicated concentrations. **B** Percent root length colonization of *L. japonicus* wild type and *ein2a ein2b* co-cultured with *R. irregularis* for 4 weeks and treated with the ethylene precursor, ethephon at indicated concentrations. Roots harvested from this experiment were utilized for RNA-sequencing. Statistics: **A** Individual data-points and mean ± SE are shown (N=6 for Solvent and 20 µM ACC, 7 for 2 µM AVG). Welch and Brown-Forsythe one-way ANOVA with Dunnett's T3 multiple comparisons test [F* (DFn, DFd) = 29.00 (2.000, 12.90) & W (DFn, DFd) = 27.11 (2.000, 9.541)] was used to assess significant differences between treatments. **B** Individual data-points and mean ± SE are shown (N=7 for 0 µM and 20 µM ethephon/WT, 6 for 0 µM ethephon/*ein2a ein2b*, 8 for 20 µM ethephon/*ein2a ein2b*). Two-way ANOVA [interaction F (1, 24) = 32.42 (P<0.0001), treatment F (1, 24) = 46.77 (P<0.0001), genotype F (1, 24) = 214.0 (P<0.0001)] with Tukey's multiple comparisons test was used to assess significant differences between treatments and genotypes. Different letters indicate statistical differences between treatments and genotypes.

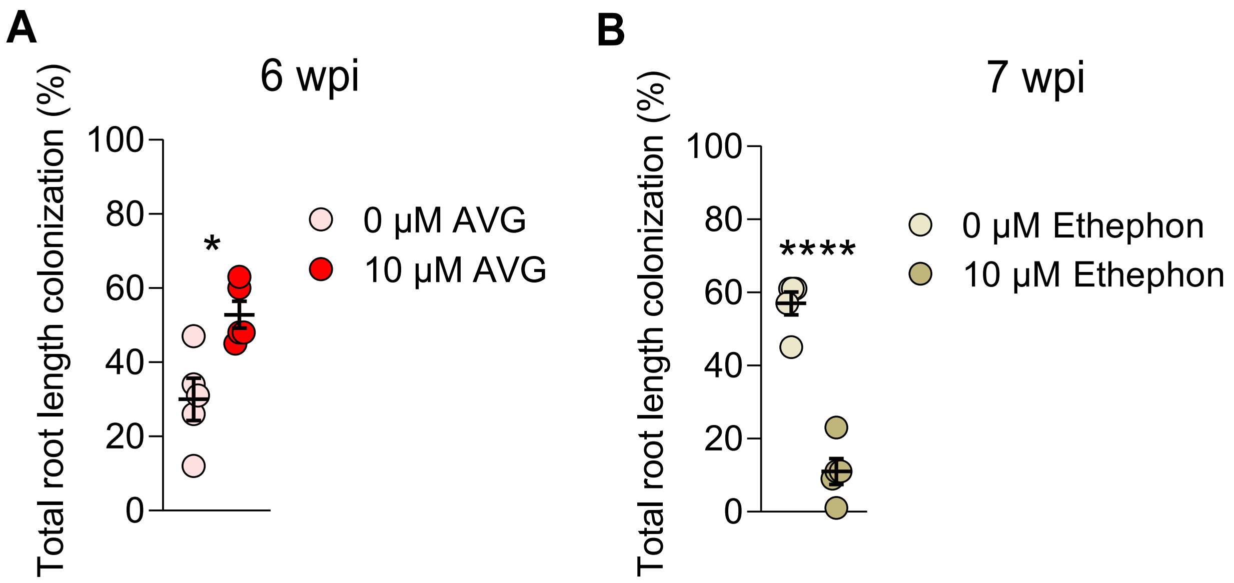

Supplementary Figure S3. Effect of ethylene on AM development in rice roots in hydroponics. A,B Percent root length colonization of rice wild type (cv. Nipponbare) co-cultured with *R. irregularis* and treated with AVG (**A**) or with ethephon (**B**) at indicated concentrations. wpi, weeks post inoculation. Statistics: **A, B** Individual data-points and mean ± SE are shown. N=5; Unpaired t test with Welch's correction (for **A**, F (DFn, Dfd) = 2.458 (4, 4) and for **B**, F (DFn, Dfd) = 1.292 (4, 4). Asterisks denote significance: * p ≤ 0.05; ** p ≤ 0.01; *** p ≤ 0.001.

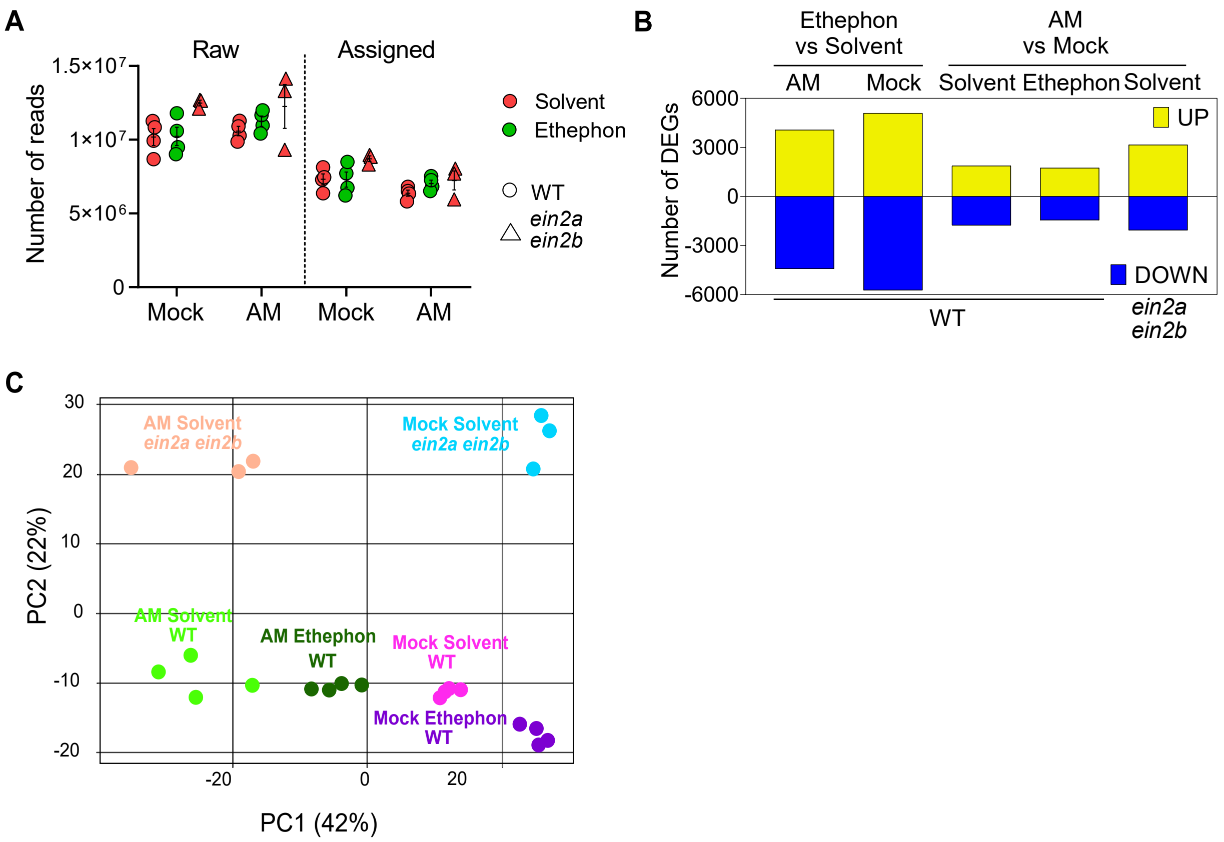

Supplementary Figure S4. Effect of ethephon treatment on the transcriptome of *L. japonicus* control and *R. irregularis*-colonized roots. A Number of RNA-Seq reads, raw or assigned to the reference genome of *L. japonicus* MG20 version 3.0, N=3 (*ein2a ein2b*) or 4 (wild type). Individual data-points and mean ± SE are shown. **B** Number of up- and down-regulated differentially expressed genes (DEGs) in ethephon (vs solvent)-dependent transcriptome of AM and mock roots and AM (vs Mock)-dependent transcriptome of ethephon and solvent treated WT and *ein2a ein2b* roots. **C** PCA plot for the transcriptome of indicated treatments and genotypes. (see also Supplementary Data S1 and S2).

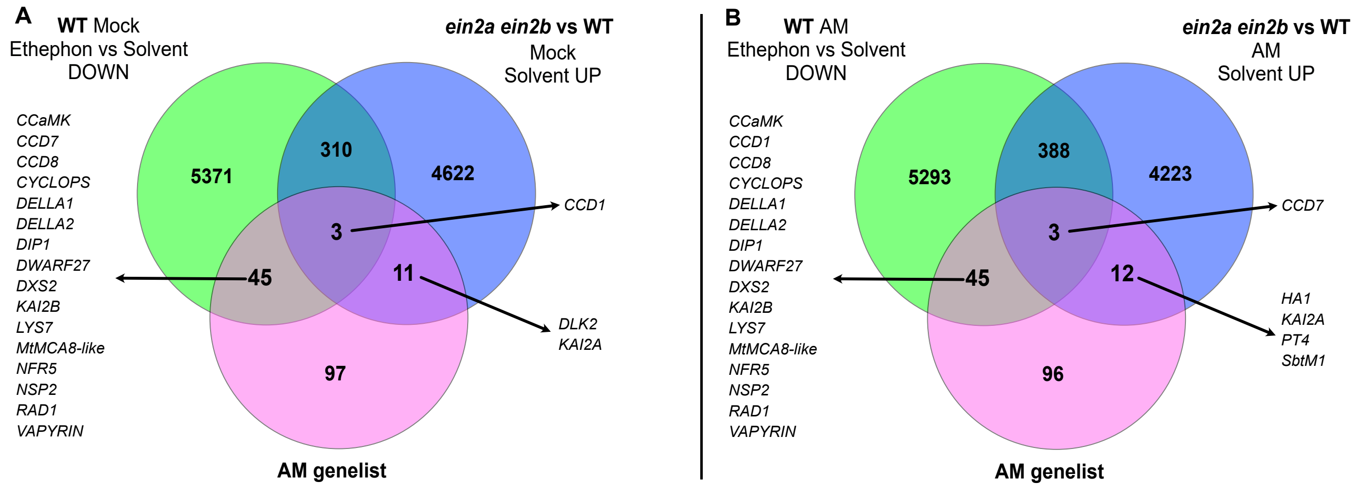

Supplementary Figure S5. A,B Venn diagrams showing overlap of DEGs with decreased transcript accumulation in wild type 'Ethephon vs Solvent'-treated roots and DEGs with increased transcript accumulation in solvent treated '*ein2a ein2b* vs WT' roots **A** non-inoculated or **B** colonized with *R. irregularis* with the AM genelist (see also Supplementary Data S5). Genes with genetically shown functions in AM are indicated.

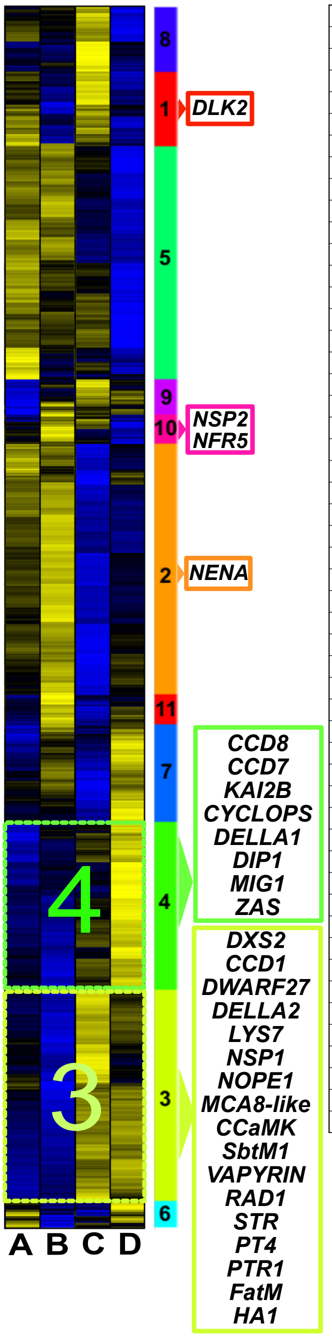

Cluster	GO term ID	GO term description	Neg$_{10}$logFDR
1	GO:0009812	flavonoid metabolic process	1.35
2	GO:0006412	translation	8.68
	GO:1901566	organonitrogen compound biosynthetic process	8.68
	GO:0043043	peptide biosynthetic process	8.52
	GO:0043604	amide biosynthetic process	7.62
	GO:0006518	peptide metabolic process	6.74
	GO:0010467	gene expression	6.66
	GO:0022613	ribonucleoprotein complex biogenesis	6.62
	GO:0042254	ribosome biogenesis	5.59
	GO:0080163	regulation of protein serine/threonine phosphatase activity	2.03
	GO:0090407	organophosphate biosynthetic process	1.74
	GO:0019538	protein metabolic process	1.47
3	GO:0006721	terpenoid metabolic process	4.40
	GO:0006720	isoprenoid metabolic process	3.16
	GO:0010623	programmed cell death involved in cell development	2.89
	GO:0006629	lipid metabolic process	2.18
	GO:0009685	gibberellin metabolic process	1.89
	GO:0051501	diterpene phytoalexin metabolic process	1.52
	GO:0009179	purine ribonucleoside diphosphate metabolic process	1.30
4	GO:0071229	cellular response to acid chemical	4.14
	GO:1901700	response to oxygen-containing compound	3.46
	GO:0071396	cellular response to lipid	2.92
	GO:0010476	gibberellin mediated signaling pathway	2.89
	GO:0009607	response to biotic stimulus	2.22
	GO:0006952	defense response	2.22
	GO:0016036	cellular response to phosphate starvation	2.21
	GO:0007154	cell communication	1.70
	GO:0010200	response to chitin	1.66
	GO:0044550	secondary metabolite biosynthetic process	1.46
	GO:2000377	regulation of reactive oxygen species metabolic process	1.44
	GO:0042743	hydrogen peroxide metabolic process	1.35
	GO:0010243	response to organonitrogen compound	1.35
5	GO:0015979	photosynthesis	4.15
6	NA	NA	NA
7	GO:0071555	cell wall organization	4.03
	GO:0005975	carbohydrate metabolic process	1.96
	GO:0006826	iron ion transport	1.54
8	GO:0019752	carboxylic acid metabolic process	1.82
9	GO:0000819	sister chromatid segregation	1.30
10	NA	NA	NA
11	NA	NA	NA

A: WT Mock Ethephon vs Solvent
B: WT AM Ethephon vs Solvent
C: WT AM vs Mock Solvent
D: *ein2a ein2b* AM vs Mock Solvent

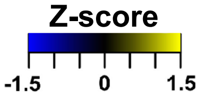

Supplementary Figure S6. Hierarchical clustering of ethephon-repressed but AM-induced genes. The heatmap shows Z-score of scaled log_2Fold-change for combined DEGs under the indicated conditions (A-D). Colored bars and accompanying numbers on the right of the heatmap depict individual clusters (based on the dendrogram on the left side). AM-relevant genes (from **Fig. 2C**) shown genetically to be functionally important for AM are mentioned on the side of clusters they belong to. Cluster-wise gene ontology (GO) enrichment analysis indicates enrichment of functional categories important for AM symbiosis such as response to gibberellin, gibberellin and lipid biosynthetic process and flavonoid metabolism in cluster-3 and -4 which contains genes suppressed by ethephon but induced by AM in WT and/or *ein2a ein2b*. To identify differentially expressed genes between groups, a two-sided exact test in edgeR, was used, accounting for both up- and downregulated genes. Adjusted p-values were calculated using the Benjamini-Hochberg procedure to control for multiple comparisons.

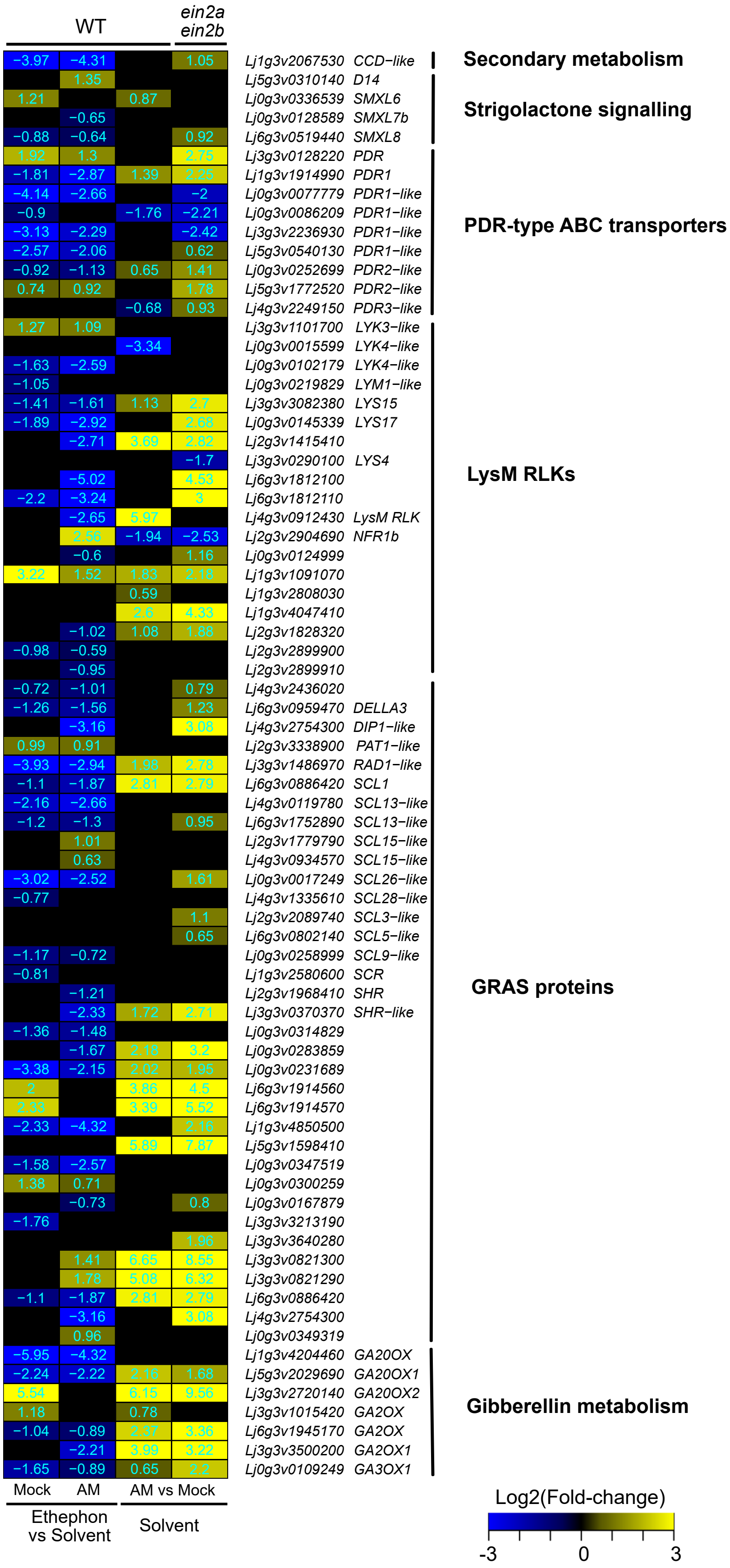

Supplementary Figure S7. Effect of ethylene signaling on RNA-Seq based expression of genes belonging to the functional categories strigolactone biosynthesis and signaling, PDR-type ABC transporters, LysM receptor-like kinases (LysM-RLKs), GRAS proteins and gibberellin metabolism. Heatmap for log_2Fold-change for genes belonging to the categories shown on the right except for genes with genetically shown functional relevance in AM (shown in **Fig. 2C**) for the indicated comparisons. To identify differentially expressed genes between groups, a two-sided exact test in edgeR, was used, accounting for both up- and downregulated genes. Adjusted p-values were calculated using the Benjamini-Hochberg procedure to control for multiple comparisons.

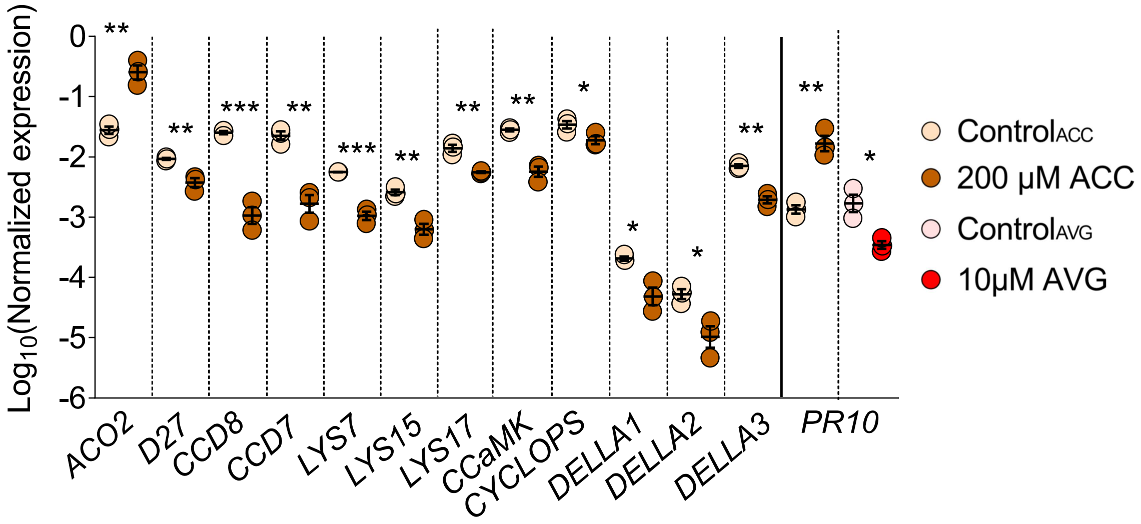

Supplementary Figure S8. Genes with reduced expression upon ethephon treatment are also reduced upon ACC treatment. RT-qPCR-based normalized transcript accumulation in roots of *L. japonicus* wild type and treated with ACC or AVG at the indicated concentrations for 4 weeks. Each unit change on the Y-axis indicates a 10-fold change in gene expression. Expression values of indicated genes were normalized to the expression of housekeeping gene, *UBIQUITIN*. Statistics: Individual data-points and mean ± SE are shown. N=3; Unpaired t-tests. * $p \leq 0.05$; ** $p \leq 0.01$; *** $p \leq 0.001$. F values for these genes were as follows (in the same order as shown in the figure): 3.803, 20.50, 4.127, 1567, 4.063, 19.40, 15.44, 1.043, 18.23, 4.658, 4.876, 3.592, 4.780. DFn, Dfd values for comparisons for each gene were 2, 2.

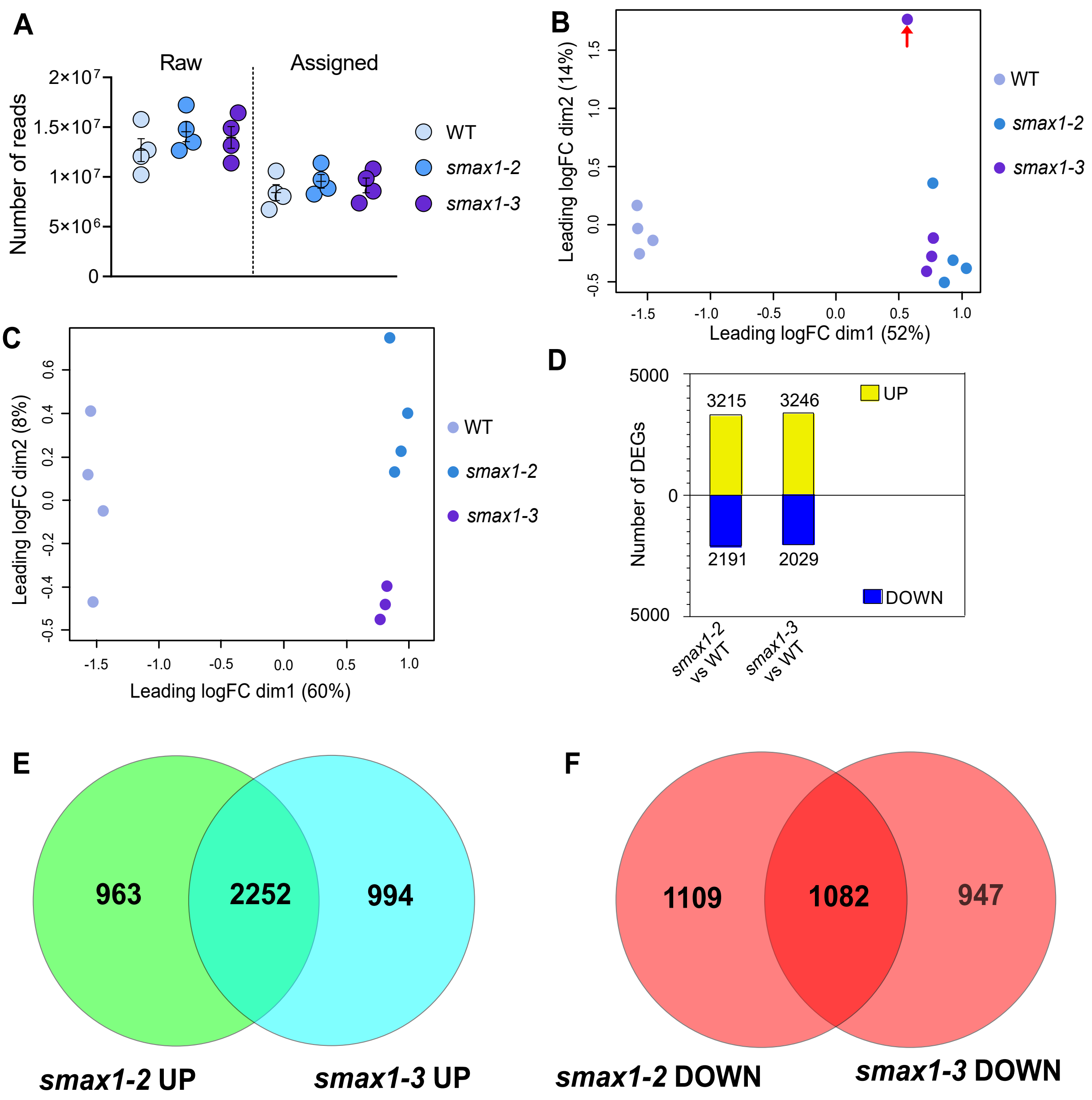

Supplementary Figure S9. Results from RNA-sequencing of *smax1-2* and *smax1-3* mutant roots. A Number of reads for *L. japonicus* wild-type, *smax1-2* and *smax1-3* mutant roots, raw and assigned to the reference genome of MG20 version 3.0. **B** Principal component analysis (PCA) for all 16 samples of wild type, *smax1-2* and *smax1-3*. Outlier is indicated by a red arrow. This outlier was removed. **C** Principal component analysis (PCA) for 15 samples of WT, *smax1-2* and *smax1-3* (excluding the outlier). **D** Number of up- and down-regulated genes in *smax1-2* and *smax1-3* vs wild type. **E-F** Comparison of *smax1-2* and *smax1-3* transcriptomes, Venn intersection of up- and down-regulated genes between the *smax1-2* and *smax1-3* mutants respectively. Only the DEGs overlapping between *smax1-2* and *smax1-3* were used for further analysis. (See also Supplementary Data S6 and Data S7).

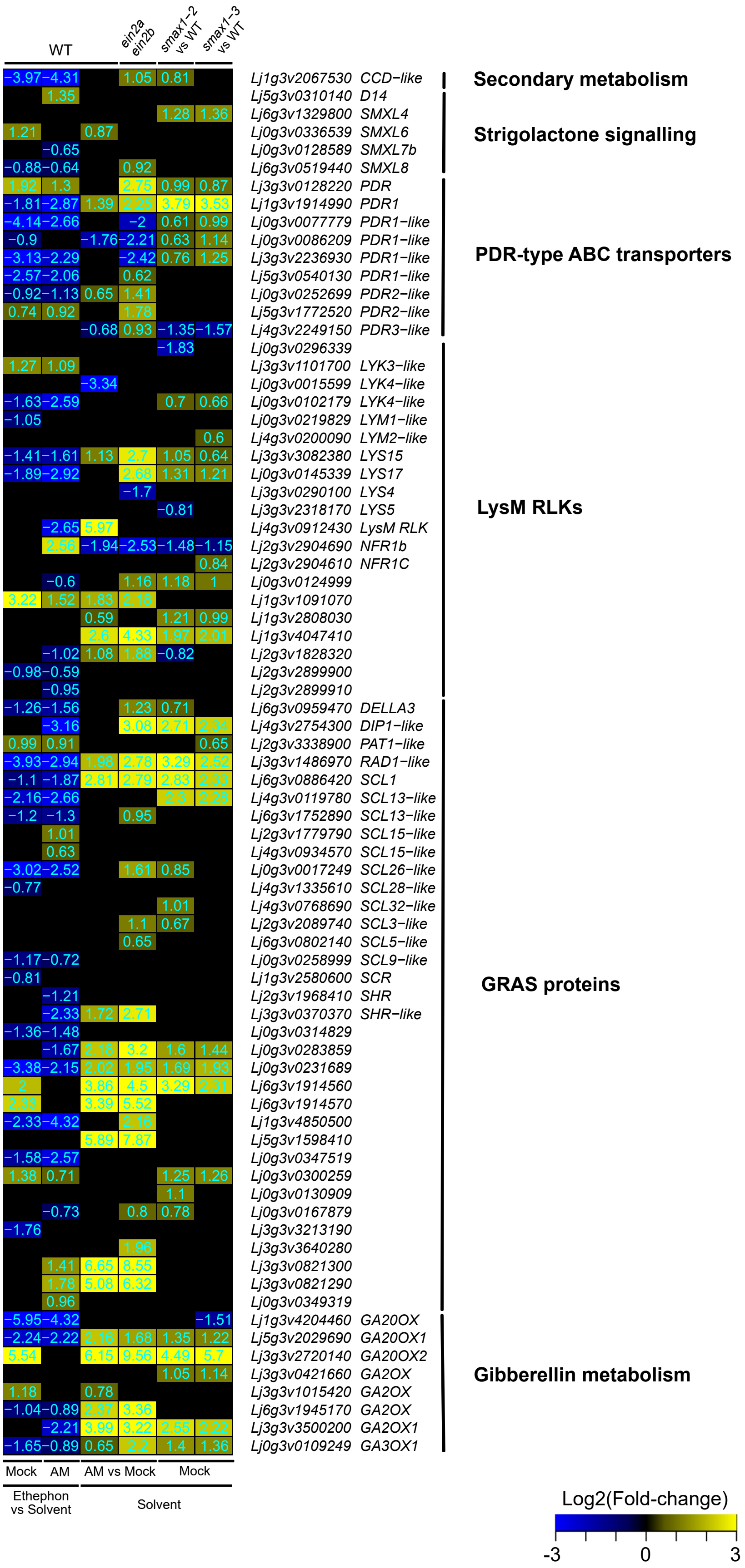

Supplementary Figure S10. Effect of ethylene signaling and *SMAX1* mutations on RNA-Seq based expression of genes belonging to the functional categories strigolactone biosynthesis and signaling, PDR-type ABC transporters, LysM receptor- ike kinases (LysM-RLKs), GRAS proteins and gibberellin metabolism. Heatmap for log_2Fold-change for genes belonging to the categories shown on the right except for genes with genetically shown functional relevance in AM (shown in **Fig. 2C, 4B-C, S5**) for the indicated comparisons. To identify differentially expressed genes between groups, a two-sided exact test in edgeR, was used, accounting for both up- and downregulated genes. Adjusted p-values were calculated using the Benjamini-Hochberg procedure to control for multiple comparisons.

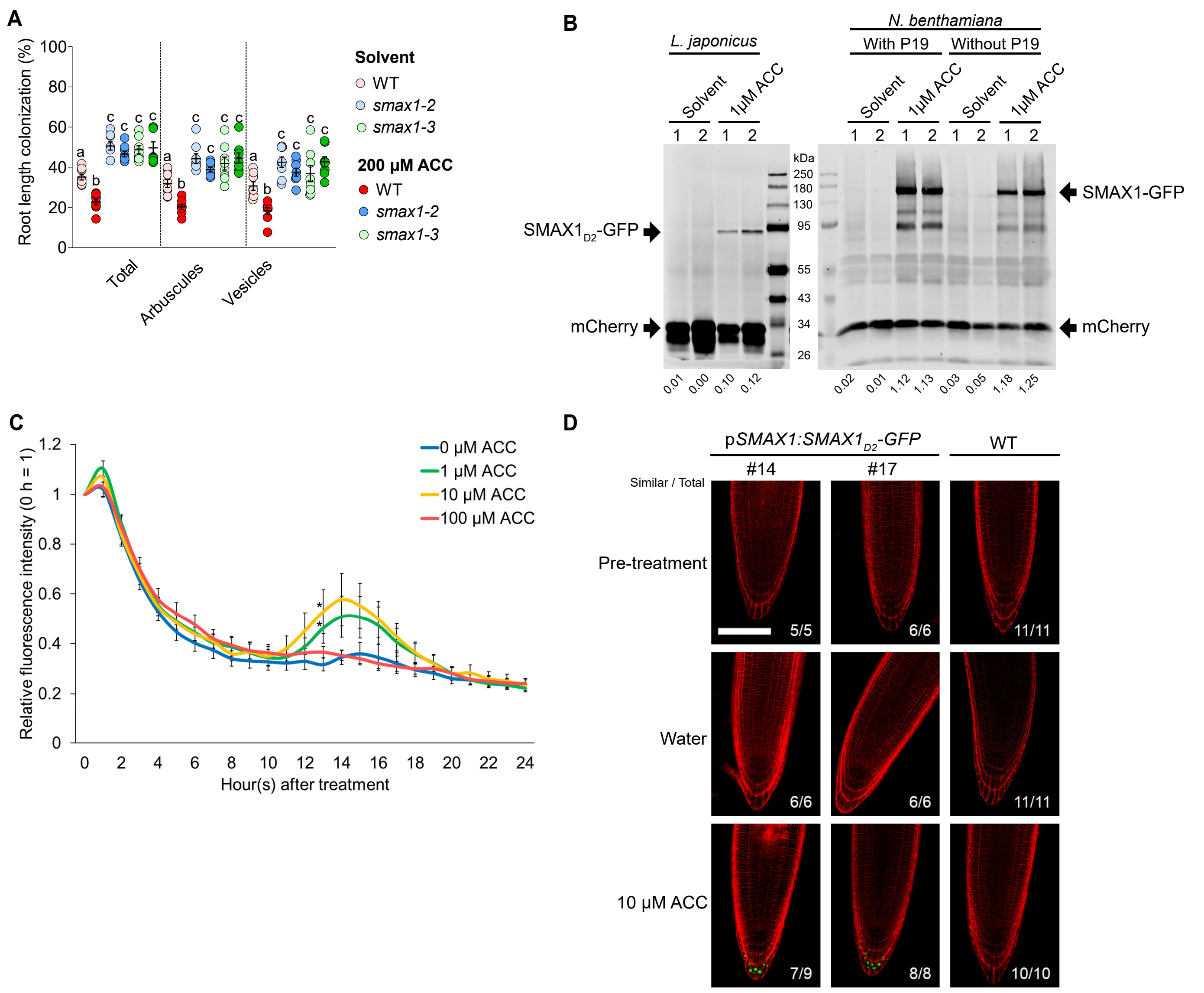

Supplementary Figure S11. SMAX1 accumulates upon ACC treatment. A Percent root length colonization of indicated *L. japonicus* genotypes with *R. irregularis* at 5 wpi and treated with the indicated chemicals. This experiment was performed independently of that shown in Fig. 5A. Individual data-points and mean ± SE (N=8) are shown. Two-way ANOVA [for total: interaction F (2, 42) =5.168 (P=0.0099), treatment F (1, 42) = 6.750 (P=0.0129), genotype F (2, 42) = 35.20 (P<0.0001); for arbuscules: interaction F (2, 42) = 5.168 (P=0.0099), treatment F (1, 42) = 6.750 (P=0.0129), genotype F (2,42) = 35.2 (P<0.0001); for vesicles: interaction F (2, 42) = 6.305 (P=0.0040), treatment F (1, 42) = 3.273 (P=0.0776), genotype F (2, 42) = 23.59 (P<0.0001)] with Tukey's multiple comparisons test was used to assess significant differences between treatments and genotypes for each indicated AMF structure. **B** Anti-GFP and anti-mCherry immunoblot of protein extracts from *L. japonicus* hairy roots expressing p*Ubi*:*LjSMAX1*$_{D2}$-*GFP* (left) and *N. benthamiana* leaf disks expressing p*Ubi*:*LjSMAX1-GFP* (right), treated with indicated chemicals for 24 hours. p*35S*:*mCherry*, expressed from the same T-DNA, was used as transformation marker. Numbers below the lanes indicate the ratio of fluorescence signal intensities of GFP-tagged proteins vs. mCherry on the original fluorescent image. Numbers above the lanes indicate independent replicates. For *Lotus* roots, the experiment was repeated independently three times with similar results. **C** Relative fluorescence intensity measured at different time points mentioned on the X-axis in 9-day-old *Arabidopsis* Col-0 seedlings expressing p*Ubi10*:*AtSMAX1*$_{D2}$-*LUC* treated in parallel with the indicated concentrations of ACC. Fluorescence is relative to time zero (set to 1). n =16. Data represent mean ± SE. *P < 0.05 (Welch's t-test, two-sided), in comparison to mock treatment for each time point. Experiments were performed three times with similar results. **D** Representative confocal microscopy images of 5-day-old root tips of indicated *Arabidopsis* genotypes. Plants were treated for 14 hours with the indicated chemicals and stained with propidium iodide before imaging. The numbers on the top left of each image represent similar phenotypes as shown per total seedlings observed. The experiment was repeated twice with equivalent results. Scale bar, 100µm.

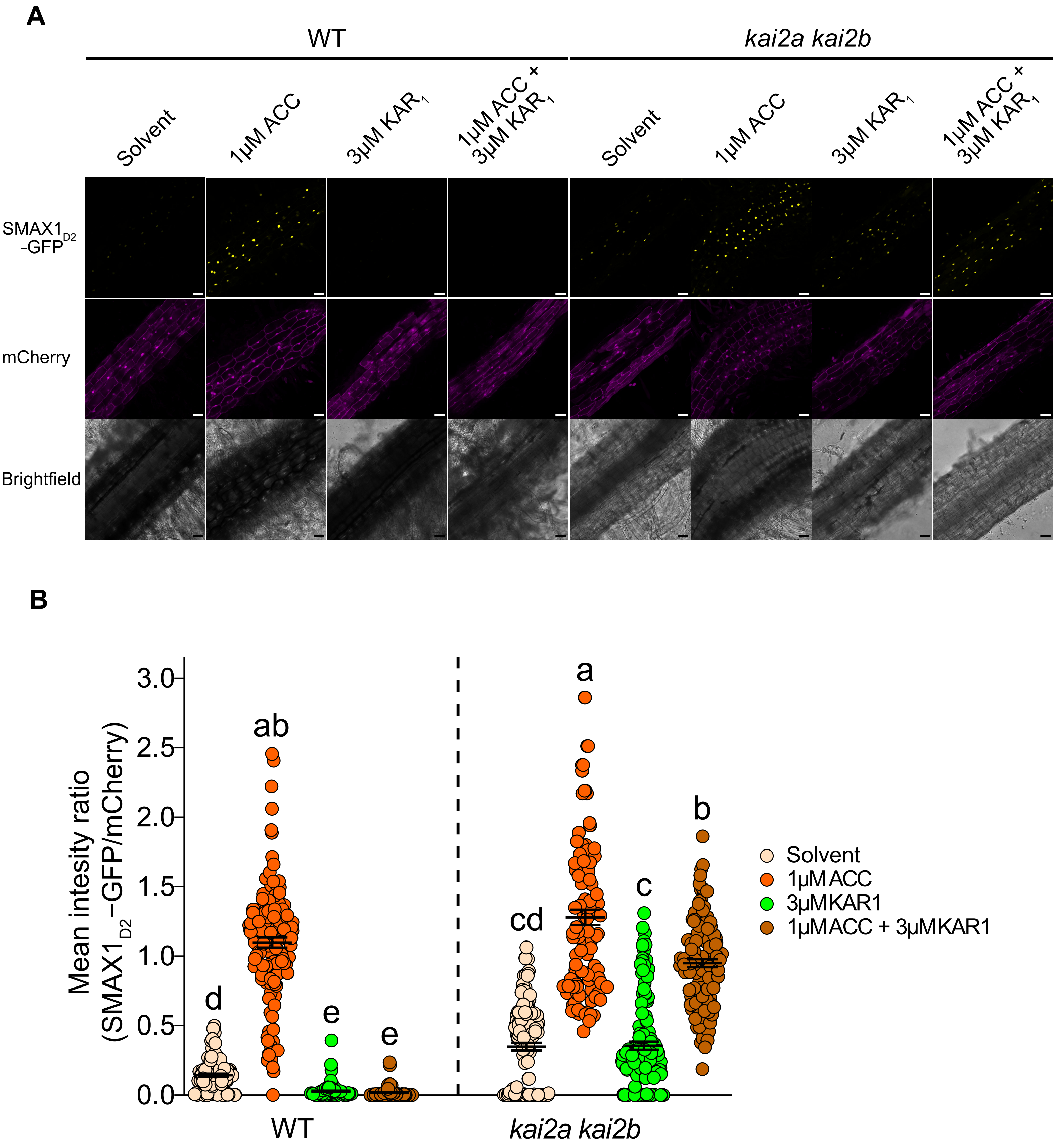

Supplementary Figure S12. KAR_1 can counteract the effect of ACC on accumulation of SMAX1 in *Lotus japonicus* in a *KAI2*-dependent manner. A Confocal microscopy images of *L. japonicus* wild-type and *kai2a kai2b* hairy roots ectopically expressing (p*Ubi*) $LjSMAX1_{D2}$ fused with GFP and a free mCherry transformation marker (p*35S*) from the same T-DNA and treated for 24 hours with solvent (0.0225% methanol solution), 1μM ACC, 3μM karrikin$_1$ (KAR_1), or a combination of both. Scale bars = 50 μm. **B** Ratios of mean intensities of nuclear GFP signal to nuclear mCherry signal measured in confocal images of hairy roots shown in **A**. Individual data-points and mean ± SE are shown; N=109 for Solvent/WT, 131 for 1μM ACC/WT, 111 for 3μM KAR_1/WT, 110 for 1μM ACC + 3μM KAR_1/WT, 125 for Solvent/*kai2a kai2b*, 107 for 1μM ACC/*kai2a kai2b*, 125 for 3μM KAR_1/*kai2a kai2b*, 117 for 1μM ACC + 3μM KAR_1/*kai2a kai2b*. Kruskal-Wallis test (Kruskal-Wallis H statistic = 642.0) with Dunn's posthoc comparison was used to assess significant differences between genotypes and treatments. Different letters indicate statistical differences.

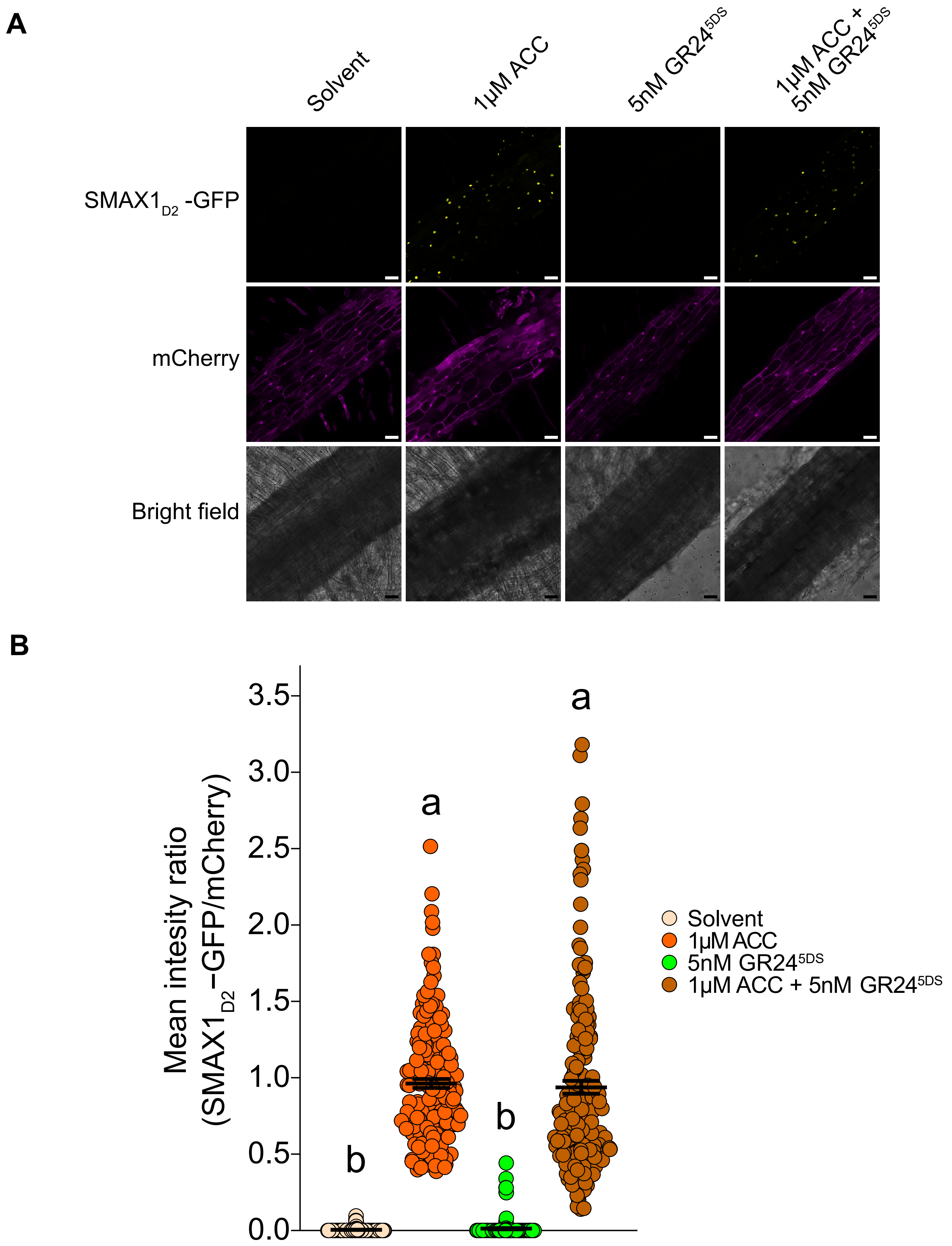

Supplementary Figure S13. Low concentration of GR24^{5DS} does not influence accumulation of SMAX1 in *Lotus japonicus*. A Confocal microscopy images of *L. japonicus* wild-type hairy roots ectopically expressing (p*Ubi*) LjSMAX1$_{D2}$ fused with GFP and a free mCherry transformation marker (p*35S*) from the same T-DNA and treated for 24 hours with solvent (0.005% acetone solution), 1μM ACC, 5nM GR24^{5DS}, or a combination of both. Scale bars = 50 μm. **B** Ratios of mean intensities of nuclear GFP signal to nuclear mCherry signal measured in confocal images of hairy roots shown in **A**. Individual data-points and mean ± SE (N=184 for Solvent, 179 for 1μM ACC, 196 for 5nM GR24^{5DS}, 190 for 1μM ACC + 5nM GR24^{5DS}) for are shown. Kruskal-Wallis test (Kruskal-Wallis H statistic = 567.0) with Dunn's posthoc comparison was used to assess significant differences between treatments. Different letters indicate statistical differences.

Supplementary Table S1. Distribution of mean intensity ratios of nuclear SMAX1$_{D2}$-GFP signal to nuclear mCherry signal shown in **Fig. 5F**. Percentage of nuclei with the given ratio are presented here.

**Intensity ratio (R)**	**WT**<br>**Solvent**<br>n = 111	**WT**<br>**1µM ACC**<br>n = 107	***ein2a-2 ein2b-1***<br>**Solvent**<br>n = 177	***ein2a-2 ein2b-1***<br>**1µM ACC**<br>n = 184
R = 0	17.11%	0%	3.39%	20.65%
0 < R < 0.5	65.77%	54.21%	84.18%	59.24%
0.5 < R < 1	10.81%	37.38%	12.43%	17.39%
1 < R	6.31%	8.41%	0%	2.72%

Supplementary Table S2. Distribution of mean intensity ratio of nuclear SMAX1$_{D2}$-GFP signal to nuclear mCherry signal shown in **Fig. S12B**. Percentage of nuclei with the given ratio are presented here.

**Intensity ratio (R)**	**WT Solvent** n = 109	**WT 1μM ACC** n = 131	**WT 3μM KAR$_1$** n = 111	**WT 1μM ACC + 3μM KAR$_1$** n = 110
R = 0	1.83%	0%	9.01%	17.27%
0 < R < 0.5	98.17%	10.69%	90.99%	82.73%
0.5 < R < 1	0%	25.19%	0%	0%
1 < R	0%	64.12%	0%	0%

**Intensity ratio (R)**	***kai2a kai2b*** **Solvent** n = 125	***kai2a kai2b*** **1μM ACC** n = 107	***kai2a kai2b*** **3μM KAR$_1$** n = 125	***kai2a kai2b*** **1μM ACC + 3μM KAR$_1$** n = 117
R = 0	3.20%	0%	12.00%	0%
0 < R < 0.5	58.40%	0.94%	64.00%	7.69%
0.5 < R < 1	37.60%	37.38%	18.40%	53.85%
1 < R	0.80%	61.68%	5.60%	38.46%

Supplementary Table S3. Distribution of mean intensity ratio of nuclear SMAX1$_{D2}$-GFP signal to nuclear mCherry signal shown in **Fig. S13B**. Percentage of nuclei with the given ratio are presented here.

**Intensity ratio (R)**	**Solvent** n = 184	**1µM ACC** n = 179	**5nM GR24^{5DS}** n = 196	**1µM ACC + 5nM GR24^{5DS}** n = 190
R = 0	25.00%	0%	40.82%	0%
0 < R < 0.5	75.00%	11.17%	59.18%	18.95%
0.5 < R < 1	0%	48.05%	0%	47.89%
1 < R	0%	40.78%	0%	33.16%

Supplementary Table S4. Primers used in this study

Cloning primers

Plasmid	Primers	
L0 Esp3I *LjSMAX1*$_{D2}$ with ATG wo STOP	KV156	ATCGTCTCACACCATGTTTGATGTATTACAGAGC
	KV157	TACGTCTCTCCTTACACTGTTCCGCCAC
L0 *LjEIN2B* CDS part 1	DD 123_CEND cds1'F_bsa	CGGGTCTCACACCATGCCAAAGGCCATACCA
	DD 116_cds1aR_bsa	ATGGTCTCGGTTGCAGAGCTACCACCACTT
L0 *LjEIN2B* CDS part 2	DD 117_cds1bF_bsa	ATGGTCTCACAACTTCCATCTTCTGTGATC
	DD 118_cds1R_bsa	ATGGTCTCGCTGCTGGGCCATCCGACG
L0 *LjEIN2B* CDS part 3	DD 119_cds2F_bsa	ATGGTCTCAGCAGCATTCAGGAGCCTTA
	DD 120_cds2R_bsa	ATGGTCTCGGCGTGGTACTTCTTAGTATT
L0 *LjEIN2B* CDS part 4	DD 121_cds3F_bsa	ATGGTCTCAACGCCTTGCCAGACATTTT
	DD 122_cds3R_bsa	GAGGTCTCGCCTTCAAGTTGTATGGTGCTGATGT

qPCR primers

Use	Primers	
qPCR *Ubiquitin* Lj5g3v2060710	Ubi F	ATGCAGATCTTCGTCAAGACCTTG
	Ubi R	ACCTCCCCTCAGACGAAG
qPCR *SbtM1* Lj2g3v2002910	SbtM1 F	CACGTTGTTAGGACCCCAAT
	SbtM1 R	TTGAGCAGCACCCTCTCTATC
qPCR *BCP1* Lj3g3v0424200	BCP1 F	TCATCTGTCCTTGGGGTCAT
	BCP1 R	CAGCTGCAGAAGTTGCATTT
qPCR *RAM1* LotjaGi1g1v0628300	Sc307	TGGAGGAAGATCATGGAAGG
	Sc308	AGCAACAAGCACCCTTTGTC
qPCR *PT4* Lj1g3v0948470	PT4 F	GAATAAAGGGGCCAAAATCG
	PT4 R	GCTGTATCCTATCCCCATGC
qPCR *AMT2.2* Lj0g3v0115479	AMT2.2 F	TGGTTCAACTTTTCGTTCCA
	AMT2.2 R	CTTATCACCCTGACCCCAGA
qPCR *ACO2* Lj6g3v1789900	DD47	TTCAAGCCATGAAGGCCGTCAA
	DD48	CAACTCTTCCTGAAGTAGATCAC

qPCR *D27* Lj1g3v4449220	DD90	TCTGCAAAATGCCATCTCAA
	DD91	GCTCGGTCCATGCTGTTTAT
qPCR *CCD8* Lj1g3v2068760	DD92	GTTCTGCCAGATGCTAAGGTTG
	DD93	GTTAGGGTTTATGCTGCACATG
qPCR *CCD7* Lj0g3v0146209	KV107	GTATGGAGTGTTTAAGATGCCC
	KV108	TAAAATGACTGCGTGGAAGC
qPCR *LYS7* Lj6g3v1812110	KV113	GCCTTGGATTCAGCAAGAGG
	KV114	AATCTGCAACCTTTCCACGG
qPCR *LYS15* Lj3g3v3082380	KV115	CAACATTGCTTGCAAGTGGAC
	KV116	TTGGTGGCCTTTTCAAGCTC
qPCR *LYS17* Lj0g3v0145339	KV117	ATGCTTTTGGAGTGGTGCTG
	KV118	AACACCACACACTTTCCCAC
qPCR *CCaMK* Lj3g3v1739280	KV105	GGAGACAATGCAACTCTGTCTGA
	KV106	CGGTGCTAGAGGGATCAATGA
qPCR *CYCLOPS* Lj2g3v1549600	KV103	GCTGGCAGATGAAAAAGAGC
	KV104	GCGTGTTTGAGCACAACATT
qPCR *DELLA1* Lj6g3v0433880	PP124	GTCCAATGGAGGACCAGGAT
	PP125	GGCAATGAGTGGCCTAGTGT
qPCR *DELLA2* Lj4g3v2436020	KV109	GTTTGGGAGGAGGAAGACGA
	KV110	TCTGATGAGAGGTGGTGAGC
qPCR *DELLA3* Lj6g3v0959470	KV111	AACGACTTGCCAAGCTGAAG
	KV112	GGCAAGCATGTTGTTAACGC
qPCR *PR10* Lj0g3v0286359	DD102	CTAAAGGTGATGCTAAACCC
	DD103	GCAAGCACTTAGAAAGAAGC

Supplementary Table S5. Golden Gate plasmids constructed in this study.

Name	Description	Cut ligation
**Golden Gate Level 0**		
L0 Esp3I *LjSMAX1*$_{D2}$	PCR amplification of *L. japonicus* Gifu cDNA with primers KV156 and KV157. Assembly into L0-pUC57 (BB02)	SmaI
**Golden Gate Level I**		
LI *gLjCCaMK*	*gLjCCaMK* with mutated BpiI and BsaI sites. Assembly into LI-pUC57 (BB03)	BpiI
LI p*SbtM1* (G84)	Assembled from: LI A-B p*SbtM1* BsaI + LI A-B dy (G84)	BsaI
LI p*PT4* (G84)	Assembled from: LI A-B p*PT4* BsaI + LI A-B dy (G84)	BsaI
LI p*Ubi* (G84)	Assembled from: LI A-B p*Ubi* BsaI + LI A-B dy (G84)	BsaI
LI p*Cor* (G84)	Assembled from: LI A-B p*Cor* BsaI + LI A-B dy (G84)	BsaI
LI p*Epi* (G84)	Assembled from: LI A-B p*Epi* BsaI + LI A-B dy (G84)	BsaI
LI EIN2B$_{CENDCDS}$ (G85)	Assembled from: PCR amplified fragment from EIN2B CDS with primers DD 123_CEND cds1'F_bsa and DD 116_cds1aR_bsa + PCR amplified fragment from EIN2B CDS with primers DD 117_cds1bF_bsa and DD 118_cds1R_bsa + PCR amplified fragment from EIN2B CDS with primers DD 119_cds2F_bsa and DD 120_cds2R_bsa + PCR amplified fragment from EIN2B CDS with primers DD 121_cds3F_bsa and DD 122_cds3R_bsa + LI C-D dy (G85)	BsaI
**Golden Gate Level II**		
LIIc F1-2 *pUbi:*GOI_*GFP* [1]	Assembled from: LI *pUbi* (G007) + LI B-C dy (BB06) + LI dy POI (G083) + LI D-E *GFP* (G011) + LI E-F nos-T (G006) + LI F-G dy (BB09) + LIIc F 1-2 (BB30)	BsaI
LIIc R3-4 p*35S:mCherry* [1]	Assembled from: LI A-B p*35S* (G005) + LI B-C dy (BB06) + LI C-D *mCherry* (G023) + LI D-E dy (BB08) + LI E-F 35S-T (G059) + LI F-G dy (BB09) + LIIc R 3-4 (BB34)	BsaI
LIIc F3-4 *pUbi:LjCCaMK*	Assembled from: LI *pUbi* (G007) + LI B-C dy (BB06) + LI *gLjCCaMK* + LI D-E dy (BB08) + LI E-F HSP-T (G045) + LI F-G dy (BB09) + LIIc F 3-4 (BB33).	BsaI
LIIc F1-2 p*Ubi:mCherry* [2]	Assembled from: LI A-B p*Ubi* (G007) + LI B-C dy (BB06) + LI C-D *mCherry* (G023) + LI D-E dy (BB08) + LI E-F 35S-T (G059) + LI F-G dy (BB09) + LIIc F1-2 (BB30)	BsaI
LIIc F3-4 p*dy*:*GOIdy-YFP*	Assembled from: LI A-B dy (G82) + LI B-C dy (BB6) + LI C-D dy (G83) + LI D-E YFP (G12) + LI E-F nosT (G80) + LI F-G dy (BB9) + LIIc F3-4 (B33)	BsaI
**Golden Gate Level III**		
LIIIβ *pUbi:*GOI_*GFP* p*35S:mCherry*	Assembled from: LIIc F 1-2 *pUbi:*GOI_*GFP* + LII 2-3 ins (BB43) + LIIc R 3-4 p*35S:mCherry* + LII 4-6 dy (BB41) + LIIIβ F A-B (BB53)	BpiI
LIIIβ *pUbi:LjSMAX1*$_{D2}$*_GFP* p*35S:mCherry*	Assembled from: LIIIβ *pUbi:*GOI_*GFP* p*35S:mCherry* + LI Esp3I *LjSMAX1*$_{D2}$	Esp3I
LIIIβ p*Ubi:LjCCaMK* p*Ubi:mCherry*	Assembled from: LIIc F 1-2 *pUbi:mCherry* + LII 2-3 ins (BB43) + LIIc F 3-4 p*Ubi:LjCCaMK* + LII 4-6 dy (BB41) + LIIIβ F A-B (BB53)	BpiI
LIIIβ p*Ubi:LjCCaMK*^{314} *p35S:mCherry* [2]	Assembled from: LII 1-3 dy (BB38) + LIIc R 3-4 p*35S:mCherry* + LII 4-5 dy (BB40) + LIIc R 5-6 p*Ubi:CCaMK*^{314} + LIIIβ F A-B (BB53)	BpiI

LIIIβ fin<br>p*Ubi:mCherry*<br>p*dy:GOIdy-YFP*	Assembled from: LII 1-2 mCherry (GC125) + LII ins 2-3 (BB44) + LIIc F3-4 p*dy:GOIdy-YFP* + LII ins 4-5 (BB43) + LII 5-6 dy (BB65) + LIIIβ fin (BB52)	BpiI
LIIIβ fin<br>p*Ubi:mCherry*<br>p*SbtM1:*EIN2B$_{CENDCDS}$-*YFP*	Assembled from: LIIIβ fin p*Ubi:mCherry* p*dy:GOIdy-YFP* + LI p*SbtM1* (G84) + LI EIN2B$_{CENDCDS}$ (G85)	Esp3I
LIIIβ fin<br>p*Ubi:mCherry*<br>p*PT4:*EIN2B$_{CENDCDS}$-*YFP*	Assembled from: LIIIβ fin p*Ubi:mCherry* p*dy:GOIdy-YFP* + LI p*PT4* (G84) + LI EIN2B$_{CENDCDS}$ (G85)	Esp3I
LIIIβ fin<br>p*Ubi:mCherry*<br>p*Ubi:*EIN2B$_{CENDCDS}$-*YFP*	Assembled from: LIIIβ fin p*Ubi:mCherry* p*dy:GOIdy-YFP* + LI p*Ubi* (G84) + LI EIN2B$_{CENDCDS}$ (G85)	Esp3I
LIIIβ fin<br>p*Ubi:mCherry*<br>p*Cor:*EIN2B$_{CENDCDS}$-*YFP*	Assembled from: LIIIβ fin p*Ubi:mCherry* p*dy:GOIdy-YFP* + LI p*Cor* (G84) + LI EIN2B$_{CENDCDS}$ (G85)	Esp3I
LIIIβ fin<br>p*Ubi:mCherry*<br>p*Epi:*EIN2B$_{CENDCDS}$-*YFP*	Assembled from: LIIIβ fin p*Ubi:mCherry* p*dy:GOIdy-YFP* + LI p*Epi* (G84) + LI EIN2B$_{CENDCDS}$ (G85)	Esp3I
LIIIβ<br>*pUbi:LjSMAX1_GFP*<br>p*35S:mCherry*	Assembled from: LIIIβ *pUbi:*GOI_*GFP* p*35S:mCherry* + LI Esp3I *LjSMAX1* A[1] + LI Esp3I *LjSMAX1* B[1]	Esp3I

Supplementary References

1. Carbonnel, S. et al. The karrikin signaling regulator SMAX1 controls *Lotus japonicus* root and root hair development by suppressing ethylene biosynthesis. *Proc. Natl. Acad. Sci. USA* **117**, 21757-21765 (2020).
2. Pimprikar, P. et al. A CCaMK-CYCLOPS-DELLA complex activates transcription of *RAM1* to regulate arbuscule branching. *Curr. Biol.* **26**, 987-998 (2016).
