# Supplementary material for: Ethylene promotes SMAX1 accumulation to inhibit arbuscular mycorrhiza symbiosis
Source: Nat Commun. 2025 Feb 27;16:2025. doi: 10.1038/s41467-025-57222-w (PMC11868565; doi:10.1038/s41467-025-57222-w)
Supplement: Supplementary file 3 — Description of additional supplementary files [file 41467_2025_57222_MOESM3_ESM.docx]

**Description of Additional Supplementary files**

Supplementary Data 1. RNA-seq based read count (for all genes) and expression data (for expressed genes) in solvent or ethephon treated mock- or AM-inoculated WT roots and in solvent treated mock- or AM-inoculated *ein2a-2 ein2b-1* roots.

Supplementary Data 2. RNA-seq based diferentially expressed genes (DEGs) in solvent or
ethephon treated mock- or AM-inoculated WT roots and in solvent treated mock- or AM-inoculated *ein2a-2 ein2b-1* roots.

Supplementary Data 3. Genes in set I-VII in the Venn diagram shown in Fig. 3A.

Supplementary Data 4. List of AM genes (AM gene list).

Supplementary Data 5. Genes in the Venn sets shared with "AM genelist" in the Venn diagram shown in Fig. S5A.

Supplementary Data 6. Genes in the Venn sets shared with "AM genelist" in the Venn diagram shown in Fig. S5B.

Supplementary Data 7. Clusters shown in Fig. S6 for the combined unique list of DEGs (12516DEGs) signifcantly regulated in Ethephon- vs Solvent-treated samples of AM- and Mock-inoculated WT roots, AM- vs Mock-inoculated solvent-treated WT and *ein2a-2 ein2b-1* roots.

Supplementary Data 8. RNA-seq based read count (for all genes) and expression data (for expressed genes) in *smax1-2* and *smax1-3* roots in comparison to WT roots.

Supplementary Data 9. RNA-seq based differentially expressed genes (DEGs) in *smax1-2* and *smax1-3* roots in comparison to WT roots.

Supplementary Data 10. Genes in the 7 Venn sets in the Venn diagram shown in Fig. 5B.
